# Production of Human papillomavirus pseudovirions in plants and their use in pseudovirion-based neutralisation assays in mammalian cells

**DOI:** 10.1038/srep20431

**Published:** 2016-02-08

**Authors:** Renate L Lamprecht, Paul Kennedy, Suzanne M Huddy, Susanne Bethke, Megan Hendrikse, Inga I Hitzeroth, Edward P Rybicki

**Affiliations:** 1 Biopharming Research Unit, Department of Molecular and Cell Biology, University of Cape Town, Rondebosch, 7701, South Africa; 2 Pharmaceutical Product Development, Fraunhofer IME, Aachen, 52074, Germany; 3 Institute of Infectious Disease and Molecular Medicine, University of Cape Town, Rondebosch, 7701, South Africa

## Abstract

Human papillomaviruses (HPV) cause cervical cancer and have recently also been implicated in mouth, laryngeal and anogenital cancers. There are three commercially available prophylactic vaccines that show good efficacy; however, efforts to develop second-generation vaccines that are more affordable, stable and elicit a wider spectrum of cross-neutralising immunity are still ongoing. Testing antisera elicited by current and candidate HPV vaccines for neutralizing antibodies is done using a HPV pseudovirion (PsV)-based neutralisation assay (PBNA). PsVs are produced by transfection of mammalian cell cultures with plasmids expressing L1 and L2 capsid proteins, and a reporter gene plasmid, a highly expensive process. We investigated making HPV-16 PsVs in plants, in order to develop a cheaper alternative. The secreted embryonic alkaline phosphatase (SEAP) reporter gene and promoter were cloned into a geminivirus-derived plant expression vector, in order to produce circular dsDNA replicons. This was co-introduced into *Nicotiana benthamiana* plants with vectors expressing L1 and L2 via agroinfiltration, and presumptive PsVs were purified. The PsVs contained DNA, and could be successfully used for PBNA with anti-HPV antibodies. This is the first demonstration of the production of mammalian pseudovirions in plants, and the first demonstration of the potential of plants to make DNA vaccines.

Human papillomaviruses (HPV) are the most common agents of viral infections of the human reproductive tract that are transmitted through sexual contact. Infection and persistence of the oncogenic high-risk HPV-type infections, such as HPV types 16 and 18, are linked to cervical cancer and other anogenital and oropharyngeal cancers in humans. The non-oncogenic or low-risk types of HPV cause common skin and genital warts and other lesions. More than a hundred HPV types have been identified of which twelve have been linked to cervical cancer^1^,^2^,^3^.

HPV is a virus with a double-stranded circular DNA genome of ~8 kb, and small non-enveloped isometric particles with a diameter of 55–60 nm. The capsid of the virus is composed of the main capsid protein L1 and the minor capsid protein L2. Although L2 is not required for capsid formation, it is thought to play a number of essential roles in viral DNA encapsidation, and in the viral infectious entry pathway to effectively deliver the viral DNA into the host cell^4^,^5^.

Two prophylactic vaccines – Gardasil (Merck) and Cervarix (GSK) - were approved by the U.S. Food and Drug Administration (FDA) in 2006 and 2009 respectively, in order to combat the development of HPV-associated cancers. These prophylactic vaccines exploit the fact that the HPV L1 self-assembles into virus-like particles (VLPs) that are both morphologically correct and highly immunogenic^6^,^7^. Merck’s second-generation vaccine Gardasil-9, approved in November 2014, is comprised of VLPs from nine different HPV types, and has the potential of preventing up to 90% of cervical, vulvar, vaginal and anal cancers.

The fact that infectious HPV virions are produced *in vivo* only in terminally differentiated keratinocytes^8^ has severely hindered studies of virus replication and vaccine development, due to a lack of an efficient and reliable way to culture the virus^9^. Testing of neutralisation of infectivity *in vivo* or *in vitro* has also been hindered: however, several methods have recently been developed to produce structurally authentic HPV pseudovirions (PsVs). It was shown that HPV VLPs produced by co-expression of L1 and L2 could package non-papillomaviral DNA *in vitro*, with similar efficiency to packaging of the papillomaviral genomic DNA. PsVs could be produced by co-transfection of L1 and L2-encoding expression vector plasmids and a plasmid encoding a reporter gene into cultured mammalian cells, and were able to transfer plasmid DNA into epithelial cells in the same manner as HPV virions would transfer the viral genomic DNA.

PsVs have since then become indispensable for their use in the pseudovirion-based neutralisation assay (PBNA), now considered the gold standard test of immunogenicity for candidate HPV vaccines. The currently-accepted method of PsV production utilises the human cell line HEK293TT for high-titre production of HPV PsVs encapsidating a secreted alkaline phosphatase-expressing (SEAP) reporter plasmid with a SV40 origin of replication to allow multiplication specifically in these cells^10^,^11^. While this is a robust and effective method of PsV production, the protocol is both highly expensive and time-consuming.

Transient expression of recombinant proteins in plants, mainly via infiltration of plants with recombinant *Agrobacterium tumefaciens* (agroinfiltration), has become a viable alternative to other more established production systems^12^,^13^. Transient expression is preferred to the establishment of transgenic plant lines as (1) it is much less time-consuming, (2) transient expression generally results in higher protein yields, (3) scale up and good manufacturing practices are adaptable, and (4) waste generated is more easily contained^13^,^14^,^15^. The development of industrial-scale vacuum infiltration equipment has shown transient expression to be a highly effective tool for large-scale production of even complex VLPs such as candidate influenza or orbivirus vaccines^16^,^17^.

Several groups have reported the successful production of papillomavirus L1 capsid proteins in plants. Both transgenic and transient expression of L1 has been done by us and by others, and spontaneous VLP assembly for HPV types 8, 11 and 16 has been shown, with varying degrees of efficiency^18^,^19^,^20^,^21^,^22^,^23^. In all instances the plant-produced VLPs were morphologically similar to VLPs produced in other systems, and elicited similar immunological responses. While expression of HPV L2 proteins is far less well studied, and plant-made L1 + L2 VLPs have not been reported in the literature, our group has successfully expressed HPV-16 L2 in *N. benthamiana* via agroinfiltration^24^.

The use of replicating DNA virus-derived vectors for transient expression in plants has recently been explored^25^,^26^. Several investigations have shown that use of geminivirus-derived vectors, and especially of Bean yellow dwarf mastrevirus (BeYDV)-derived vectors, is a successful strategy for high-level protein production for products as diverse as candidate vaccine proteins or whole monoclonal antibodies^25^,^27^,^28^.

In this study, we investigated the use of plants to manufacture HPV-16 PsVs, in order to develop a significantly cheaper and easier alternative for producing PsVs. To achieve this, we modified our previously-developed self-replicating BeYDV-derived pRIC3.0 vector plasmid to provide the reporter gene which would be expressed in mammalian cells. The plant-produced PsVs were purified, and were tested by means of the PBNA for their similarity to mammalian cell-made particles, and for whether or not they had encapsidated the plant-made replicon DNA. This study has demonstrated for the first time the successful production and effective neutralisation by antibodies of known activity of plant-produced HPV PsVs tested in parallel with conventionally-produced PsVs, and has expanded the potential of developing reagents and vaccines under the ever-expanding plant molecular farming technology.

## Results

### Constructs produced SEAP in mammalian cells

**Figure 1:**
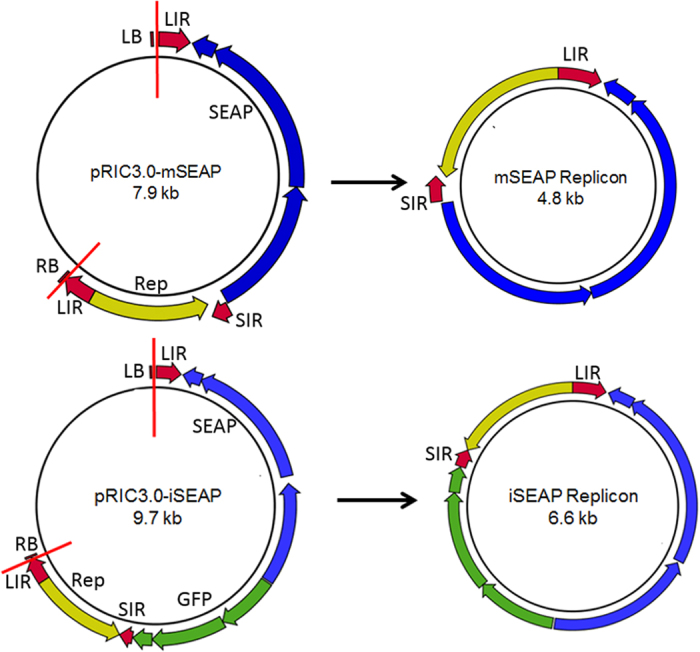
The pRIC3.0-mSEAP and pRIC3.0-iSEAP constructs and their replicons. The top panel shows the pRIC3.0-mSEAP construct that recircularizes into the mSEAP replicon after the release of the T-DNA and the bottom panel shows the pRIC3.0-iSEAP construct. LIR, BeYDV long intergenic region; SIR, BeYDV short intergenic region, LB, left border; RB, right border, Rep (yellow), BeYDV *rep* gene; red vertical and semi vertical lines, the relative position where the T-DNA is transfected into the plant cells. The vector recircularizes at the duplicated LIR. The blue elements indicate the mammalian expression cassette and the green elements indicate the plant expression cassette.

In order to develop constructs to be used as the reporter genes for the PsVs, the self-replicating plant expression vector pRIC3.0 was modified by inserting a mammalian expression cassette that includes the secreted embryonic alkaline phosphatase (SEAP) gene. Two constructs were developed, pRIC3.0-mSEAP and pRIC3.0-iSEAP, with the first containing only a mammalian expression cassette, and the latter containing both mammalian and plant expression cassettes. Autonomous replication of the constructs in plants would result in replicons of 4.8 kb and 6.6 kb for mSEAP and iSEAP, respectively (Fig. 1), a size range within the size limit (<8 kb) to be packaged by the co-expressed HPV capsid proteins. HEK293TT cells were transfected with either 1 μg of pRIC3.0-mSEAP or pRIC3.0–iSEAP plasmid DNA to determine if these constructs were able express SEAP in mammalian cells. SEAP was readily detected by western blot analysis at 3 dpi (data not shown). This verified that the modified plant expression vectors could be used as the reporter genes in the PsVs.

### Production and purification of the plant-produced HPV PsVs

**Figure 2:**
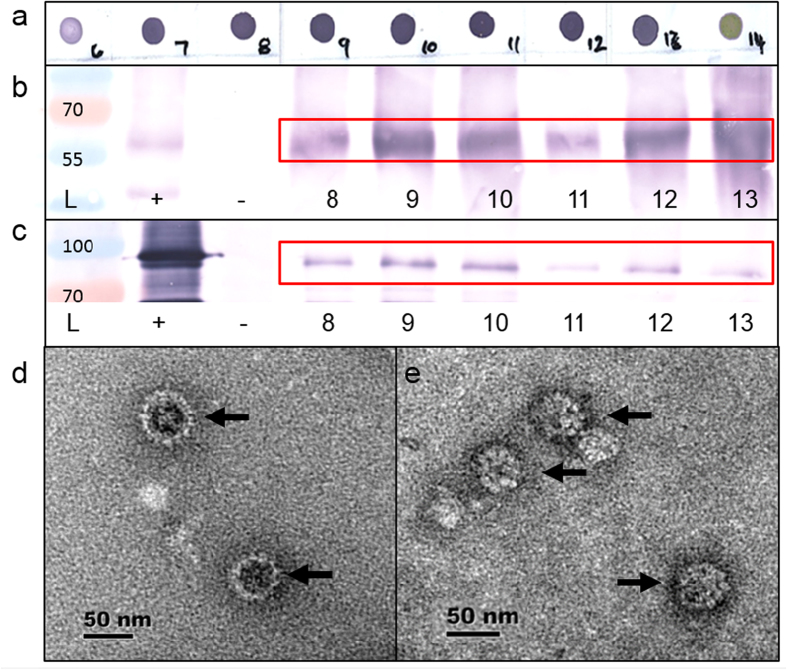
Detection of PsVs in (**a**) dot blots of fractions 6–14, (**b**) L1 western blots of fractions 8–13, (**c**) L2 western blots of fractions 8–13 and (**d**) electron micrographs of HEK293TT cell produced PsVs and (**e**) electron micrographs of plant-produced PsVs. The red boxes (in **b,c**) indicate the expected L1 and L2 sizes on the western blots, and the black arrows (in **d,e**) indicate the HPV particles. L, PageRuler Prestained Protein Ladder (Life Technologies) with sizes indicated; +, L1 or L2 positive controls, respectively, −, plant negative control.

Production of HPV PsVs has characteristically been accomplished by co-transfecting the capsid proteins of HPV together with a SEAP reporter plasmid in HEK293TT cells^10^,^11^. Based on some of these principles, we produced HPV PsVs transiently in plants by use of agroinfection. *Nicotiana benthamiana* plants were co-infiltrated with *Agrobacterium tumefaciens* harbouring constructs that contained the human codon optimised L1 and L2 in the pTRAc plant expression vectors to form the HPV capsid, and either pRIC3.0-mSEAP or pRIC3.0-iSEAP where the resulting replicon was to be encapsidated by the HPV capsid proteins. After 4 days, infiltrated leaves were harvested and subjected to ultracentrifugation on sucrose and Optiprep gradients. Presence and localisation of the HPV PsVs were observed as dark purple dots in L1 dot blots (Fig. 2a), which were mostly concentrated in fractions 7–13 that corresponded to the 33% layer of the gradient. These fractions were further analysed by western blots, transmission electron microscopy (TEM), PCR and pseudo-infection of mammalian cells.

Fractions were denatured and investigated by western blot analysis using anti-L1 and anti-L2 antibodies (Fig. 2b,c). Both L1 and L2 were detected in all fractions of interest. No signal for L1 and L2 in dot- and western blots was detected for the upper low-density phase of the Optiprep gradient, which demonstrates the efficacy of the purification method (data not shown).

To determine whether the expressed proteins had in fact formed intact particles, fractions of interest were examined by TEM. The mSEAP and iSEAP preparations were indistinguishable from each other, with a range of particle sizes from 30–120 nm. The particles showed a similar morphology throughout the 33% layer of the gradient and to other examples of plant-produced HPV L1-only particles^20^,^23^. Particles could be detected in all of the fractions representing the 33% layer of the gradient (Fig. 2d,e).

### Plant-produced pseudovirions successfully encapsidate pseudogenome DNA *in planta*

**Figure 3:**
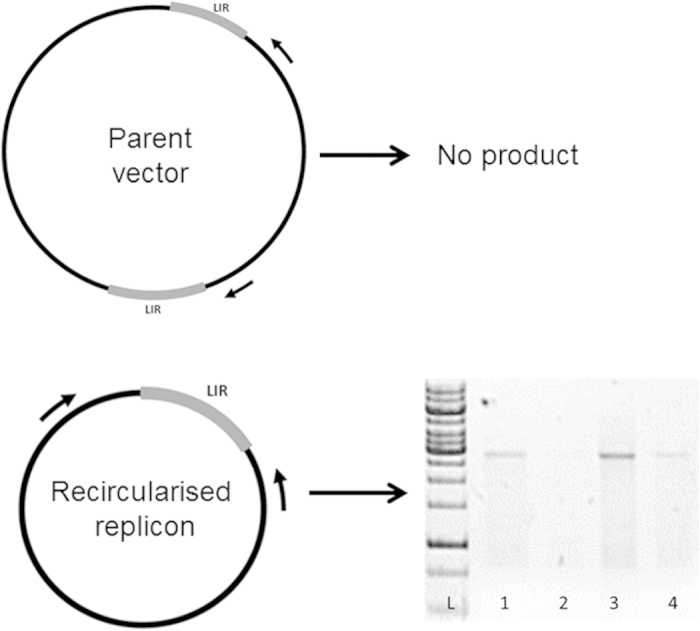
Presence of replicon DNA in purified PsVs. The diagram illustrates that a PCR product will only be present if recircularization of the replicon occurred. LIR, long intergenic region; arrows indicate primer positions. The bottom right part of the picture indicate the PCR products resulting from the inverse PCR; L, GeneRuler 1 kb DNA Ladder (Life Technologies); 1, mSEAP fraction 8 with proteinase K; 2 mSEAP fraction 8 without proteinase K; 3, mSEAP fraction 13 with proteinase K; 4, mSEAP fraction 13 without proteinase K.

To confirm that replicon DNA was encapsidated in the PsVs, L1-containing fractions were either digested or not digested with proteinase K to release the encapsidated nucleic acid, followed by PCR with replicon-specific primers (Fig. 3). PCR amplification confirmed that the PsVs contained either the mSEAP or iSEAP replicons, with the presence of the 2.1 and 2.3 kb bands, respectively. A clear difference in the intensity of the bands between the proteinase K-treated and non-treated samples were seen, as the digested samples yielded considerably more DNA than the undigested samples. This is an indication that most of the replicon DNA was encapsidated by the HPV capsids *in planta* prior to being purified.

However, the proteinase K may be responsible for removing PCR inhibitors as well as nucleases, and therefore a benzonase treatment would be a better indication for encapsidation of DNA. Prior to HEK293TT cell PsV pseudoinfection, both HEK293TT- and plant-produced PsVs were treated with benzonase and the resulting SEAP readings were compared between the benzonase-treated and non-treated groups. The SEAP readings of the benzonase-treated plant-made PsVs dropped only by 29.1% and 29.7%, for the 0.1% and 0.3% benzonase treatments, respectively. For the similarly-treated HEK293TT-produced PsVs, the SEAP readings dropped by 28.5% and 31.1%, respectively. This provides evidence that the plant-produced PsVs were equivalent to the HEK293TT-produced PsVs in terms of protecting pseudogenome DNAs, and that the SEAP readings were a result of the encapsidated pseudogenome, and not free DNA in the samples.

### Plant-produced HPV16 pseudovirions are neutralised by HPV antibodies

**Figure 4:**
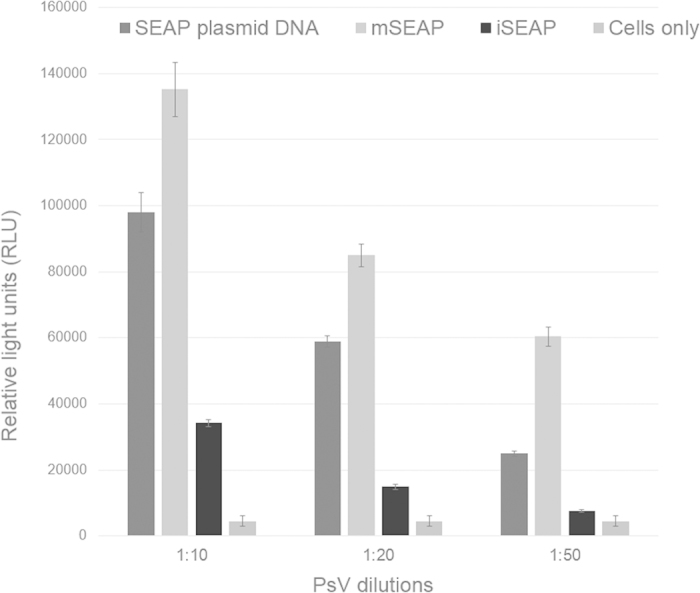
The plant-produced PsVs were able to produce SEAP after pseudo-infection of mammalian cells. SEAP expression was detected 3 days after HEK293TT cells were pseudo-infected with either SEAP plasmid DNA, plant produced mSEAP or iSEAP PsVs. Readings from untransfected/-infected cells were used as a baseline. Readings were done in triplicate. Error bars are indicated.

HEK293TT cells were initially infected with diluted samples of fractions 8–13 to determine the relative positions of empty capsids and/or PsVs in the density gradient. The SEAP readings of fractions 8 and 9 had the similar readings as the uninfected cells, while the SEAP readings for fractions 12 and 13 were at least three times higher (depending on the batch) than that of the lowest fractions in the 33% layer of the gradient. In every experiment we consistently observed that the SEAP levels produced by the iSEAP PsVs were lower than the mSEAP PsVs, and therefore only mSEAP PsVs were used for PBNA (Fig. 4)

**Figure 5:**
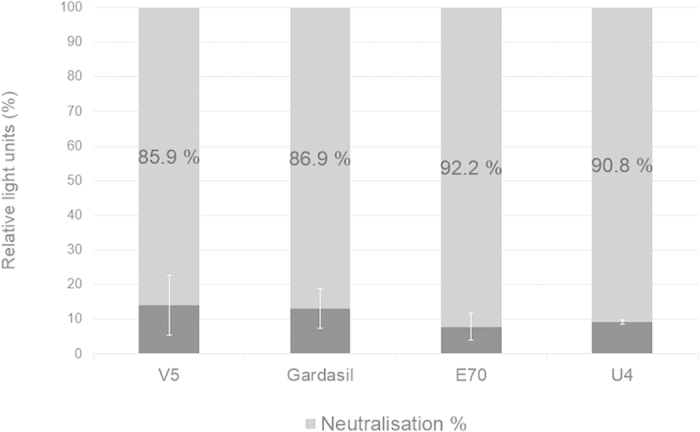
Pseudovirion-based neutralisation assay using the plant-produced PsVs. Plant-made PsVs were pre-incubated with HPV-16 neutralising antibodies V5, E70, U4 and Gardasil antiserum and then used to infect HEK293TT cells for use of PBNA. Uninfected cells were used as a baseline and neutralizing activity was expressed as a percentage of neutralization compared to that of the average RLU readings of PsVs without antibody, which was set at 100%. The lighter shade of grey represents the SEAP signal that was reduced in the presence of the specific antibody, while the darker shade of grey represents the remaining SEAP signal post neutralisation. Error bars are indicated.

For PBNA, HEK293TT cells were pseudo-infected purified PsVs, pre-incubated with or without HPV16 L1-specific neutralising antibodies V5, E70, U4 and anti-Gardasil antiserum. As seen in Fig. 5, a decrease in reporter activity was observed for plant-produced PsVs in the presence of the antibodies. An 85.9–98% reduction in signal was observed when mSEAP PsVs were pre-incubated with V5, 86.9–95.1% neutralisation for Gardasil, 92.2–97.6% neutralisation for E70 and 90.8–91.7% neutralisation in the presence of the U4 antibody. These neutralisation rates were comparable to the neutralisation ratios seen by the HEK293TT-produced PsVs (Supplementary Fig. 1): in the presence of the V5 antibody the HEK293TT-PsVs SEAP signal was reduced by 88.2–93.1%, with Gardasil 86.1–91.5%, with E70 86.8–95.3% and with U4 the SEAP signal was reduced by 44.7–71.8%. All PBNA experiments were repeatable from different batches of purified PsV stocks.

## Discussion

The past decade has seen excellent progress towards the control of new HPV infections with the roll-out of HPV prophylactic vaccines. Unfortunately, as the available vaccines against HPV are very type-specific, it is still necessary to develop vaccines against HPV that would protect against the other types that cause cancer. All of these candidate vaccines would then have to be assessed for their effectiveness in producing neutralising antibodies. For this reason, the PBNA was developed and was shown to be an efficient approach for the detection of neutralising antibodies^29^. More recently the technology has evolved to develop HPV PsVs that encapsidate SEAP-encoding plasmids, which is now considered to be the gold standard in high-throughput papillomavirus neutralisation assays^11^.

Another recent and more exciting application of PsVs was in delivery of a potential DNA vaccine or gene therapy agent *in vivo*. Subcutaneous vaccination of mice with HPV-16 PsVs encapsidating an ovalbumin (OVA)-encoding DNA vaccine elicited significantly stronger CD8+ T cell immune responses than naked DNA vaccination via gene gun, and PsVs infected bone marrow-derived dendritic cells *in vitro*^30^. A follow-up showed that OVA PsVs could elicit therapeutic responses to OVA-expressing tumours in mice, as well as generating stronger OVA-specific CD8+ T cell responses than DNA delivered by other delivery methods^31^, indicating its utility as a therapeutic vaccine delivery system. A later study showed that intravaginal vaccination of macaques with several types of HPV PsVs containing SIV Gag DNA elicited Gag-specific humoral and T-cell responses that allowed a memory response following intravaginal challenge using SIVmac251^32^. This demonstrates a significant potential for HPV-derived PsVs to be used as DNA delivery vehicles, whether for use as vaccines or potentially for gene therapy.

However, production of HPV PsVs in mammalian cells is very expensive. The costs of PsV production could potentially be cut down significantly, however, if they were to be produced in a different expression system, such as in plants. Producing HPV PsVs in plants may also be safer than the conventional technique, as HPV capsids package DNA promiscuously, and making HPV PsVs in HEK293TT - which stably expresses the SV40-T large antigen, a known proto-oncogene - is not that favourable for safety reasons. This risk and that of other potentially infective DNA contamination is almost nil if PsVs are made and DNA packaged in plants.

In this study, we exploited a prolifically-replicating geminivirus-based plant expression vector for the production of the DNA pseudogenome to be encapsidated by HPV capsid proteins. These vectors rely on a rolling circle replication mechanism^26^ to amplify the copy number of a transgene by several orders of magnitude, with the aim of improving on transgene expression compared to non-replicating vectors. The use of such a vector is a cheaper and more efficient and elegant solution to produce a pseudogenome, than the delivery of a non-replicating pseudogenome vector for intracellular assembly, or the *in vitro* disassembly-reassembly of VLPs in the presence of bacterially-synthesised pseudogenome DNA.

SEAP is widely used as a robust and reliable reporter gene in mammalian systems, and one of the major advantages of using SEAP is that the alkaline phosphatase is secreted into the cell medium, and cell lysis is therefore not required before detection. Recombinant SEAP is also extremely heat-stable, and endogenous alkaline phosphatase will not be detected using well-established SEAP detection kits. Both the pRIC3.0-mSEAP and –iSEAP self-replicating vectors produced appreciable SEAP levels in mammalian cells. The plant gene present in pRIC-iSEAP was introduced into the construct purely to increase the size of the final replicon from 4.8 to 6.6 kb, as the efficiency of DNA packaging increases when the plasmid is close to the original size of the HPV genome^11^,^33^. The functionality of the plant gene in pRIC3.0-iSEAP was therefore not tested. There is also no evidence to suggest that the presence of the plant expression cassette had any influence on SEAP production in the mammalian cells.

Both the HPV L1 and L2 capsid proteins are required for efficient packaging of DNA into the HPV virion, in both natural virions and PsVs^34^,^35^,^36^. The presence of L2 in the PsV capsid increases DNA packaging efficiency 10-fold^5^. Furthermore, attempting to produce PsVs in the absence of L2 would result in non-infectious VLPs, as L2 facilitates the binding and delivery of encapsidated DNA into the host cell. Accordingly, L1 and L2 were co-expressed in plants in this study to allow for maximum potential DNA encapsidation. This is incidentally the first report in a journal of successful production and incorporation of HPV L2 into VLPs in plants.

Electron micrographs clearly demonstrate the successful assembly of virion-like particles resulting from L1/L2 co-expression in plants. The particles demonstrated an unusual variability in size when compared to other VLP and PsV production methods^20^,^33^,^37^. This is probably for the same reason that HPV L1-only VLPs appear to be rather pleomorphic, with a similar range of sizes, when made in plants compared to those made in mammalian or insect cells: the high concentration of L1 in plant cells may lead to less specificity in assembly intermediates than is the case in other systems^18^,^20^.

Previous studies on the production of HPV PsVs have used benzonase treatment coupled with PCR to demonstrate that DNA is encapsidated within the virion shell, and not merely associated with the virion^38^,^39^. Initially, we found that PsVs produced in plants were apparently not degraded by the 95 °C PCR denaturation step, and this resulted in little-to-no amplification of pseudogenome DNA in PsV samples. Accordingly, we introduced a proteinase K digestion step before PCR, which dramatically increased amplification of a pseudogenome-specific target. However, for reasons previously specified, we also did a benzonase treatment of both the HEK293TT- and plant-produced PsVs prior to pseudo-infection of HEK293TT cells, and compared the resulting SEAP readings between PsV preparations and to results obtained without benzonase treatment. A similar albeit small decrease in SEAP readings was observed in HEK293TT cells infected with both PsV preparations, indicating that the plant-produced PsVs protected pseudogenomes against benzonase as well as the HEK293TT-produced counterparts. We can therefore confidently conclude, from the data from both inverse PCR and benzonase treatment experiments, that the pseudogenome DNA was within the particles and not on their surfaces or free in the medium. These results also demonstrate the stability of the plant-made PsVs.

While we regard the production of PsVs derived from a human virus *in planta* as an achievement in itself, it is only relevant if the plant-produced PsVs can be used for delivery of pseudogenome DNA into mammalian cells. To test this, the plant-made PsVs were used to pseudo-infect mammalian cells. From the start of the pseudo-infection experiments, the SEAP readings from the iSEAP PsVs were consistently lower than the SEAP levels produced by mSEAP: this was an unexpected result, as we expected the iSEAP to be packaged more efficiently into the HPV capsids as it is more similar in size to the HPV genome, whereas mSEAP is almost half the size of the HPV genome. It is possible that the small size of mSEAP allowed more replicons to be packaged and resulted in higher SEAP readings than iSEAP. Different sizes of SEAP-variant replicons will be studied in the future. Although essentially any plasmid under 8 kb in size can be packaged by L1 and L2, PsV production efficiency varies with different reporter plasmids for reasons that are not fully understood. This was also seen previously in other studies^11^. Despite the different RLU readings from different batches of mSEAP and iSEAP PsVs, a reduction of between 86.6–97.6% in reporter signal was observed in repeated experiments, indicating successful neutralisation by well-known antibodies. These antibodies were chosen as they have shown high neutralisation efficiencies with the conventionally-made PsVs in our hands as well as in other studies^40^,^41^.

In conclusion, this study has demonstrated for the first time the successful production of HPV PsVs in plants. Particles were easily purified, were stable at high temperatures, and were functionally indistinguishable from PsVs produced in the conventional system. To our knowledge, this is the first demonstration of this approach for HPV or any other animal virus. The use of a vector DNA derived from a plant virus that replicates in plants to produce a pseudogenome, which is also encapsidated in the plant by co-expressed animal virus proteins that are able to infect mammalian cells, is an elegant, inexpensive and highly novel method for HPV PsV production. The system could also feasibly be extended to polyomavirus PsVs, given the recent demonstration that transient plant expression of PyV VP1 also efficiently produced VLPs that were capable of encapsidating nucleic acid, albeit non-specifically^42^.

While this is a preliminary proof-of-concept study only, it demonstrates that transient plant-based production of HPV PsVs is a highly feasible strategy. Moreover, for the first time it demonstrates that plant-made DNA expression vectors may be used in mammalian cells, which opens up the possibility of making encapsidated DNA vaccines in plants - which could become a highly attractive prospect for future exploitation for both animal and human therapeutic and prophylactic vaccines. It is also likely that plant production of PsVs intended for use as DNA delivery vehicles may be far more easily scalable to high levels than the transfection of cultured cells that is presently the mode of choice, given the ease of use of agroinfiltration for even large numbers of plants^43^.

## Methods

### Constructs

The autonomously replicating BeYDV-derived pRIC plasmid was described previously^25^. Due to recombination concerns, several repeated sequences (scaffolding and promoter regions) were removed, a multiple cloning site was introduced, and the new plasmid was re-named pRIC3.0 (R Ogle, EP Rybicki, II Hitzeroth, unpublished work). To provide the reporter gene plasmids required for the PBNA, pRIC3.0 was modified to include a mammalian gene expression cassette for the expression of SEAP. Two replicating vectors were made for this purpose: for pRIC3.0-mSEAP the original plant gene expression cassette was replaced with the mammalian gene expression cassette to produce a 4.8 kb replicon; and pRIC3.0-iSEAP contained both a mammalian and plant gene expression cassette to produce a 6.6 Kb replicon. In both cases the SEAP reporter gene is under control of the elongation factor 1 alpha (EF-1α) promoter and the simian virus 40 (SV40) polyadenylation signal. Additionally, to generate high expression of pseudogenomes in mammalian HEK293TT cells, an SV40 origin of replication (SV40-ori) was inserted into the pRIC3-mSEAP and pRIC3-iSEAP constructs, as it is thought that replication will enhance reporter gene expression levels^11^.

### Growth and maintenance of HEK293TT cells

HEK293TT cells were used for all mammalian expression experiments. Cells were grown in Dulbecco’s Modified Eagle Medium (DMEM), with 10% fetal calf serum (FCS, v/v), 1% nonessential amino acids (NEAA, v/v), 1% penicillin/streptomycin (v/v) and 250 μg/ml hygromycin. Cells were maintained in T75 cell culture flasks (Corning), and incubated at 37 °C, with 5% CO_2_, and 95% humidity. Cells were passaged when they reached 90% density, with a seeding density of 10% (approximately 1 × 10^5 ^cells ml^−1^).

### Reporter gene expression testing in HEK293TT cells

In order to verify activity of the mammalian reporter cassette, endotoxin-free plasmid DNA was used to transfect HEK293TT cells with FuGENE 6 transfection reagent (Promega) according to the manufacturer’s instructions. Transfected cells were incubated for 72 hours and western blot analysis was used to detect SEAP expression in the transfected HEK293TT cells. Cell media was harvested and heat-denatured in the presence of SDS- loading buffer (250mM Tris-Cl pH6.8, 500mM DTT, 10% SDS, glycerol and bromophenol blue) and separated on a SDS-Page gel. Proteins were transferred to a nitrocellulose blotting membrane (GE Healthcare Life Sciences) and probed with sheep-produced calf-intestinal alkaline phosphatase (AP) polyclonal antibody at 1:10000 dilution (Abcam ab7330) and detected by mouse anti-sheep AP conjugated secondary antibody (Sigma A8062).

### Pseudovirion production in plants

To produce HPV16 PsVs in *N. benthamiana* plants, several plant expression vectors were utilised. In addition to pRIC3.0-mSEAP and –iSEAP, the plant expression vector pTRAc^20^ (Prof. Rainer Fischer; Fraunhofer Institute for Molecular Biology and Applied Ecology, Aachen, Germany) containing either HPV-16 L1 or L2 human codon-optimised genes (pTRAc-hL1 and pTRAc-hL2, respectively) were used for production of L1 and L2 capsid proteins. This vector targets L1 and L2 expression to the cytoplasm and pTRAc-hL1 has demonstrated high transient expression levels for L1 *in planta*^20^.

*Agrobacterium tumefaciens* strain GV3101::pMP90RK containing the respective plant expression vectors was grown over two days and diluted to specific optical densities and co-infiltrated into 6–8 week old *N. benthamiana* plants, by submerging whole leaves into 1 L of bacterial suspension in a vacuum chamber. The leaves were subjected to a vacuum of −95 kPa and the vacuum was rapidly released (approximately 10 kPa/s) as soon as the desired vacuum was reached. Plants were grown as described^20^ and harvested at 4 dpi.

For comparison reasons, standard mammalian PsVs were also produced and purified from HEK293TT cells as previously described^10^,^11^.

### Pseudovirion purification and detection

PsVs were harvested at 4 dpi; western blotting was used to confirm the presence of L1 and L2 protein, and inverse PCR was used to confirm that replicational release had taken place. Leaves were harvested and homogenised in 1:2 (w/v) high salt phosphate buffer (HS-PBS, with 0.5 M NaCl) containing EDTA-free protease inhibitor (Roche). The plant extract was placed on ice for 30–60 min, filtered through a two layers of Miracloth (Merck Millipore), and centrifuged twice at 10 000g for 15 minutes. The supernatant was layered onto a double sucrose cushion (50 and 30%) and centrifuged at 32000 rpm for 1:15 hours in an Optima™ L-100 XP centrifuge (Beckman Coulter) with a Beckman Coulter SW32Ti rotor. The 30% layer was collected and pooled after centrifugation followed by overnight dialysis in HS-PBS. The samples were layered on top of a 50-40-33-20% Optiprep gradient (Sigma-Aldrich) and centrifuged for 4 hours at 32 000 rpm. The gradients were fractionated (20 fractions of ~800 μl) using a Foxy Jr. Fractionator (ISCO). Presence of L1 in each fraction was confirmed by dot blots by probing with CamVir anti-L1 antibody (Abcam) at a dilution of 1:20 000. Fractions containing high amounts of L1 (as indicated by dark purple dots) were further analysed with L1/L2 western blots, electron microscopy and PCR.

### Western blots

For western blotting, fractions of interest were denatured together with SDS-PAGE loading buffer and equal amounts of protein were separated by SDS-PAGE. Proteins were transferred onto a nitrocellulose membrane as described^20^. L1 was detected with CamVir (1/20 000) primary monoclonal antibody (Abcam, ab69) and goat anti-mouse AP-conjugated (1/10 000) secondary antibody (Sigma-Aldrich, A3562). L2 was detected with our own rabbit-produced anti-L2 (1/5 000) primary polyclonal serum and goat anti-rabbit (1/10 000) AP-conjugated secondary antibody (Sigma-Aldrich, A3687).

### Electron microscopy

For both the plant- and HEK293TT-made PsVs, fractions that produced the darkest dot blots were diluted in HS-PBS and trapped on carbon-coated copper grids and stained with 2% uranyl-acetate. The samples were viewed at 27 000–50 000 magnification with a Technai F20 transmission electron microscope.

### Inverse PCR

Inverse PCR was used to confirm the presence of the reporter gene replicon inside the PsVs. A PCR amplification-product will only be produced in the presence of a recircularised replicon (Fig. 3). DNA was extracted from the fractions in either the presence or absence of proteinase K (protK) for PsV capsid digestion. Fractions were mixed with PBS in a 2:3 ratio with protK or not. The samples were incubated at 55 °C for 3 h followed protK inactivation at 90 °C. The samples where cleaned using QIAquick PCR Purification Kit (Qiagen) according to the manufacturer’s instructions. Primers Rep_For (5′-TCCATCGTGCGTCAGATTTGCG-3′) and SEAP_Q (5′-GGCTCTGTCCAAGACATACAATGTA-3′) were used for replicon amplification. The presence of 2.1 or a 2.3 kb product confirms the presence of either the mSEAP or iSEAP replicons, respectively.

### Benzonase treatment of PsVs

Both the HEK293TT- and plant-produced PsVs were treated with benzonase prior to being added to HEK293TT cells for pseudo-infection. A 100 μl aliquot of purified plant-produced or HEK293TT-produced PsVs were mixed with 400 μl full DMEM and treated with either 0.1% or 0.3% (w/v) of ultrapure Benzonase (Sigma-Aldrich) and incubated at room temperature for 1 hour. After the 1 hour incubation the benzonase-treated PsVs were added to pre-seeded HEK293TT cells and incubated at 37 °C, with 5% CO_2_ and 95% humidity. SEAP levels were read 3 days later, using the Great EscAPe SEAP Chemiluminescence Kit (Clontech Laboratories) according to the manufacturer’s instructions. The samples were read in triplicate, and compared to HEK293TT cells infected with plant- or cell-made PsVs that had had no benzonase treatment.

### Pseudo-infection of plant-produced PsVs and Pseudovirion-based neutralisation assay

Initially, the L1 positive fractions were analysed for SEAP production in HEK293TT cells, to establish where in the gradient the PsVs accumulate. A 100 μl of the fractions were mixed with 400 μl full DMEM and dropped onto 150 000 cells, after which SEAP production was detected using the Great EscAPe SEAP Chemiluminescence Kit (Clontech Laboratories) 3 days later according to the manufacturer’s instructions. Cells transfected with plasmid DNA (pSEAP2, included in the Great EscAPe SEAP Chemiluminescence Kit, Clontech Laboratories) was used as positive controls. Uninfected cells were used for RLU baseline readings. SEAP readings were considered positive if the RLU readings were at least twice the RLU amount of uninfected cells. The highest SEAP producing fractions were used for PBNA.

For PBNA HEK293TT cells were seeded at a density of 150 000 cells/ml in full DMEM growth medium and plated in 25 well plates and incubated overnight. The following day the cells were pseudo-infected either with pseudovirions only, or with pseudovirions with HPV16 neutralising antibodies (PsV-Ab) V5, E70 and U4 (kindly provided by Neil Christensen (Department of Pathology, College of Medicine, Pennsylvania State University, Hershey, PA) and anti-Gardasil rabbit antiserum made by injecting rabbits with Gardasil. For the plant-produced PBNA, 100 μl of the highest SEAP-producing fractions were either mixed with 100 μl of 1:200 diluted antibody (final dilution of 1:1000) or full growth medium, to a final volume of 500 μl. For comparison reasons HEK293TT-produced PsVs were also made as described previously^10^,^11^ and were included in the PBNA with the same antibodies. The PsV-Ab and PsVs were placed on ice for an hour and added to the cells. The plates were incubated for 3 days at 37 °C, with 5% CO_2_, and 95% humidity, after which SEAP production was detected using the Great EscAPe SEAP Chemiluminescence Kit (Clontech Laboratories) according to the manufacturer’s instructions. Each sample was transferred to a 96 well plate, in triplicate, and measured using a Modulus Microplate Reader (Promega). Neutralizing activity was expressed as a percentage of neutralization compared to that of the average RLU readings of PsVs without antibody, which was set at 100%.

## Additional Information

**How to cite this article**: Lamprecht, R. L. *et al.* Production of Human papillomavirus pseudovirions in plants and their use in pseudovirion-based neutralisation assays in mammalian cells. *Sci. Rep.* **6**, 20431; doi: 10.1038/srep20431 (2016).

## Supplementary Material

Supplementary Information
