# Supplementary material for: Production of Human papillomavirus pseudovirions in plants and their use in pseudovirion-based neutralisation assays in mammalian cells
Source: Sci Rep. 2016 Feb 8;6:20431. doi: 10.1038/srep20431 (PMC4745065; doi:10.1038/srep20431)
Supplement: Supplementary Information [file srep20431-s1.pdf]

Production of Human papillomavirus pseudovirions in plants and their use in pseudovirion-based neutralisation assays in mammalian cells

Renate L Lamprecht[1], Paul Kennedy[1], Suzanne M Huddy[1], Susanne Bethke[2], Megan Hendrikse[1], Inga I Hitzeroth[1,*], and Edward P Rybicki[1, 3]

Supplementary Figure 1:

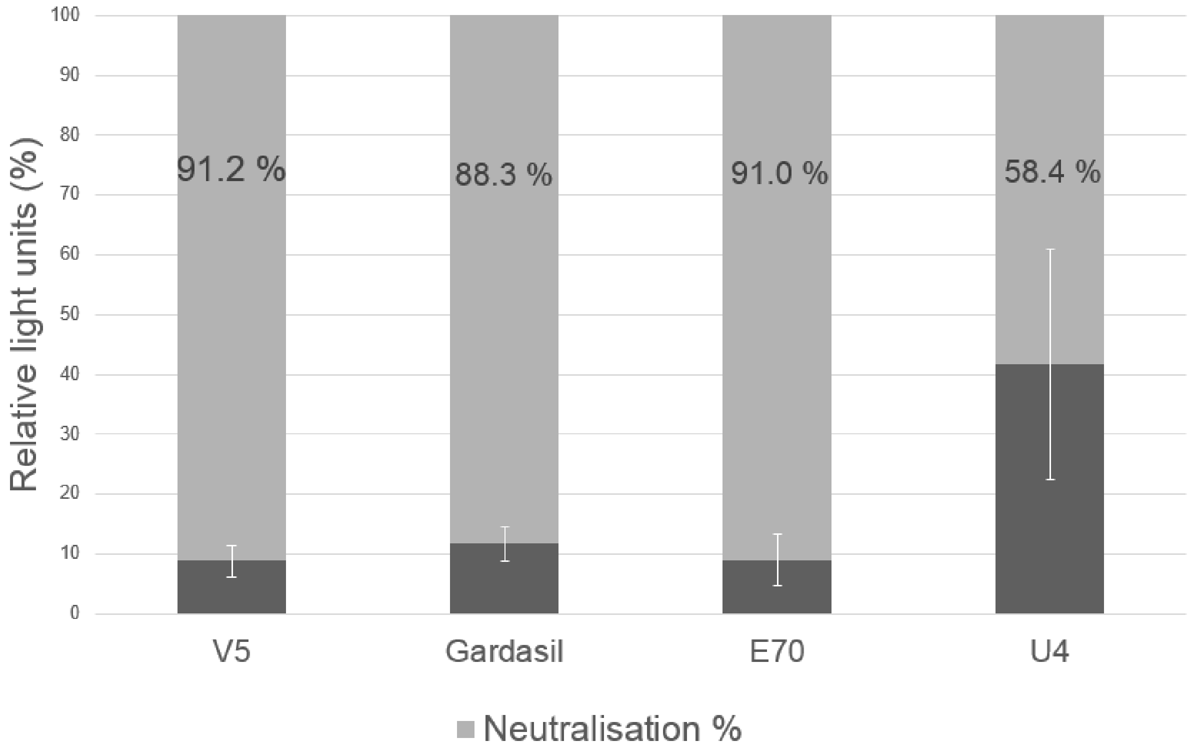

Supplementary Figure 1: Pseudovirion-based neutralisation assay using the Hek293TT-produced PsVs. Hek293TT-produced PsVs were pre-incubated with HPV-16 neutralising antibodies V5, E70, U4 and Gardasil antiserum and then used to infect Hek293TT cells for use of PBNA. Uninfected cells were used as a baseline and neutralizing activity was expressed as a percentage of neutralization compared to that of the average RLU readings of PsVs without antibody, which was set at 100%. The lighter shade of grey represents the SEAP signal that was reduced in the presence of the specific antibody, while the darker shade of grey represents the remaining SEAP signal post neutralisation. Error bars are indicated.
